# Developing a written action plan for children with eczema: a qualitative study

**DOI:** 10.3399/bjgp17X693617

**Published:** 2017-12-05

**Authors:** Kingsley Powell, Emma Le Roux, Jonathan P Banks, Matthew J Ridd

**Affiliations:** Centre for Academic Primary Care, School of Social and Community Medicine, University of Bristol, Bristol.; Centre for Academic Primary Care, School of Social and Community Medicine, University of Bristol, Bristol.; National Institute for Health Research Collaborations for Leadership in Applied Health Research and Care West (NIHR CLAHRC West), University Hospitals Bristol NHS Foundation Trust, Bristol, UK.; Centre for Academic Primary Care, School of Social and Community Medicine, University of Bristol, Bristol.

**Keywords:** atopic eczema/dermatitis, child, eczema, primary care, qualitative research, self-management, written action plans

## Abstract

**Background:**

Eczema is common in children but adherence to treatments is poor. Written action plans (WAPs) have been shown to help in asthma but the potential value, format, and content of an eczema WAP is unknown.

**Aim:**

To explore the potential role of an eczema WAP, and to design an eczema-specific WAP.

**Design and setting:**

A qualitative study of parents of children with eczema, primary and secondary care health professionals, and other stakeholders.

**Method:**

A total of 41 semi-structured one-to-one interviews and two focus groups were audiorecorded, transcribed, and analysed thematically.

**Results:**

Reported challenges of managing eczema included: parental confusion about treatment application; lack of verbal and written advice from GPs; differing beliefs about the cause and management of eczema; re-prescribing of failed treatments; and parents feeling unsupported by their GP. An eczema WAP was viewed as an educational tool that could help address these problems. Participants expressed a preference for a WAP that gives clear, individualised guidance on treatment use, presented in a step-up/step-down approach. Participants also wanted more general information about eczema, its potential triggers, and how to manage problem symptoms.

**Conclusion:**

An eczema WAP may help overcome some of the difficulties of managing eczema, and support families and clinicians in the management of the condition. Further evaluation is needed to determine if the eczema WAP the authors have developed is both acceptable and improves the outcomes for affected children and their families.

## INTRODUCTION

Eczema is a common condition in children, characterised by dry skin and intermittent flares. In the UK, the majority of children with eczema can be effectively treated in primary care with topical treatments: emollients and topical corticosteroids (TCS).^1^ However, regimes to moisturise the skin and manage flares can be complex and challenging for parents in terms of knowing when and how to apply treatments, as well as managing other factors that can exacerbate eczema.^2^ Failure to use topical treatments correctly is common^3^ and is the main cause of poor clinical outcomes,^1^^,^^4^^–^6 with negative effects on the quality of life of the child and their family.^7^^,^^8^

Research with patients and carers has identified barriers to effective treatment, including lack of understanding about the condition and treatments,^8^^,^^9^ and reluctance to use TCS because of concerns about side effects.^4^^,^^10^ Written action plans (WAPs) are patient- or carer-held instructions to support self-management. They have the potential to address these problems and improve outcomes for affected children and their families,^8^ and their use is advocated by national guidelines.^1^^,^^11^

WAPs have been shown to improve clinical outcomes in asthma,^12^^–^14 which is also common in children and requires a high degree of self-management. However, research into self-management of eczema has only identified two trials of WAPs, both of limited methodological quality.^15^ Furthermore, eczema WAPs have not been subject to prior developmental work with respect to acceptability, structure, or content.

The aims of this study were to work with health professionals, patients, and stakeholders to explore the perceived value of an eczema WAP, and to develop a WAP that could be used to support eczema self-management in primary care.

## METHOD

The authors undertook 41 semi-structured interviews and two focus groups with parents of children with eczema, GPs, and a range of other clinicians and stakeholders between May 2016 and February 2017. They focused on children under 12 years with mild to moderate eczema because these represent the majority of eczema cases in primary care, and because the needs of adolescents and adults are likely to be different.^1^

### Sampling and recruitment

The authors identified five socioeconomically diverse practices in and around Bristol, UK. Each practice searched its electronic medical records and sent an invitation letter to around 100 randomly selected parents of children aged <12 years who had an eczema diagnosis. Primary care healthcare professionals (GPs, practice nurses, and health visitors) were recruited at participating practices and via personal and professional networks.

**How this fits in**

Incorrect use of topical treatments is common in children with eczema, and contributes to poor clinical outcomes. Written action plans (WAPs), drawn up between healthcare professionals and parents and children, could support the self-management of eczema. The parents, GPs, and other healthcare professionals in this study felt that a WAP could improve parents’ knowledge and confidence in using and applying prescribed treatments. Using interviews and focus groups, the authors developed an eczema WAP, comprising individualised treatment guidance, generic eczema information, and a treatment log.

Parents and healthcare professionals expressed interest in taking part in interviews and/or focus groups by completing an online or paper questionnaire, and their responses were used for purposive sampling. Parents were sampled by socioeconomic status (calculated from their home postcode), age, sex, ethnicity of their child with eczema, and self-rated eczema severity (POEM: patient-oriented eczema measure).^16^ Older children, at the discretion of the parent, were also invited to take part in the interview. GPs were sampled by sex, years in job role, sociodemographic area of practice, and experience of using, and their perceived value of, WAPs.

Other stakeholders included secondary care clinicians (dermatologists, dermatology nurses, and allergy consultants), who were identified by snowballing and professional networks, and eczema charity representatives, school nurses, and nursery staff, who were identified via eczema charities and local school networks.

#### Interviews

In-depth interviews enabled the authors to collect data on the individual experiences and challenges of managing and treating eczema from the perspective of carers and healthcare professionals, as well as the elements that participants would find helpful in a WAP. They also provided the opportunity for participants to give feedback on the initial drafts of the WAP, which the authors developed and refined iteratively alongside the interviews. The interviews were held in participants’ homes, on NHS premises, or by telephone, and lasted 45–60 minutes. A semi-structured topic guide was developed for each participant group (that is, parents, clinicians, and stakeholders) and piloted (Box 1). Topics were modified over the course of the data collection in response to emergent topics of interest.

Box 1. **Topic guide framework**

**Table:** 

**Interviews**	**Focus groups**
Experience of managing eczema Barriers and facilitators to eczema self-management Previous use of WAPs Perceived value of WAPs Content and design preferences of the WAP Critique of example or draft WAPs Completing, sharing, and updating WAPs Training and/or resources needed to support use of WAPs	Thoughts on findings to date Critique of draft WAP Factors to consider for implementation of WAP

*WAP* = *written action plan.*

One of the authors, a GP with a specialist interest in dermatology, conducted the majority (13/15) of GP interviews. Another author, a non-clinical researcher, interviewed other participants. To facilitate discussion, early interviewees were shown three example WAPs, which varied in length, layout, and graphical content. Two were eczema-specific WAPs, one developed by the Hillingdon Hospitals NHS Foundation Trust, and the other by the Australasian Society of Clinical Immunology and Allergy (ASCIA). The third was a WAP for patients with asthma developed by Asthma UK. Sample WAPs were replaced in later interviews with successive drafts of the study WAP.

### Focus groups

Focus groups were employed to further refine, and reach a consensus around, the draft study WAPs. The first group was held towards the end of data collection, when the majority of the individual interviews (*n* = 35) had been conducted, and the second group was the final part of data collection, when data saturation from one-to-one interviews had been reached. Representatives were recruited from across the different participant groups to each focus group to promote a balanced discussion. For the first focus group discussion (FGD1), the authors invited primary care healthcare professionals (GPs, pharmacists, and eczema charity representatives) and parents who had been interviewed, because they understood the research aims and lived locally enough to travel to the university premises where the groups were held. For the second focus group discussion (FGD2), all those who had taken part in the first focus group were asked again, with the invitation being extended to a further four GPs, nine parents, and local secondary care healthcare professionals who had expressed interest in taking part, or who had already participated in an interview.

Each focus group lasted 1.5 hours. One of the authors, an academic GP with a research interest in eczema, led the discussion, ensuring all views were heard. Another observed and took notes. A separate topic guide was used for each group (Box 1).

#### Transcription and end of data collection

Groups and interviews were audiorecorded, transcribed verbatim, and anonymised. Data collection ended at the point of data saturation, when new insights related to the research questions ceased to emerge.^17^

### Data analysis

Transcripts were imported into QSR NVivo (version 10). Data were analysed thematically.^18^ Two authors used the broad requirements of the WAP to deductively develop codes, but were also open to inductively identifying codes and themes that could enhance the acceptability of the developing WAP. To maximise rigour, eight interview transcripts were coded by two authors independently and then compared to assess coding agreement, resolve any discrepancies, and identify new emergent codes. One author then coded the remaining transcripts for the primary care healthcare professionals, and the other coded the remainder of the parent, stakeholder, and secondary care healthcare professional transcripts. The same codes were applied to the interviews and focus groups. Codes within and between participant groups were compared and grouped into themes and sub-themes.

The authors developed and refined an eczema WAP over the course of the study. The final draft was produced with the assistance of a professional graphic designer, to maximise its visual appeal and usability.

### Ethics

All participants were given written information about the study, and written informed consent (or assent for children) was received prior to the interview or focus group. All participants were nominally reimbursed for their time.

## RESULTS

### Participant recruitment and characteristics

**Figure 1.**
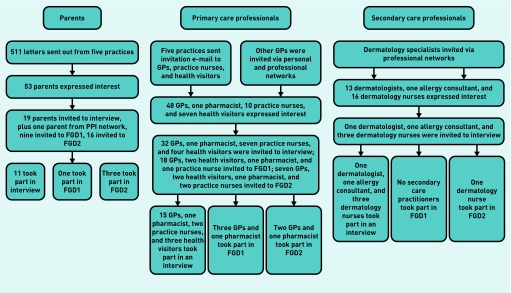
***Flowchart of recruitment pathway for parents and healthcare professionals. FGD1 = focus group discussion 1. FGD2 = focus group discussion 2. PPI = patient and public involvement.***

Figure 1 shows the recruitment process for parents and healthcare professionals (*n* = 37). The four other stakeholders were self-selected from the organisations approached. Table 1 describes interview and/or focus group participants. In all, 13 parents, two children, 24 primary care healthcare professionals, six secondary care healthcare professionals, and four stakeholders took part. Participating parents’ children ranged from 0–11 years, with POEMs between 1–24 (that is, clear/mild to severe eczema),^19^ and varied ethnicity (three black, one mixed race, nine white). The authors identified three main themes: the challenges of managing eczema, eczema WAP acceptability, format, and content, and finalising the WAP.

**Table 1.** Participant characteristics^a^

**Participant group**	**Interview participants**	**Focus group participants**	
		**Focus group 1**	**Focus group 2**
Parent	11	1 (1)	3 (1)**[1]**
GP	14	3 (1)	2 (2)**[1]**
GPwSI	1	0	0
Practice nurse	2	0	0
Health visitor	3	0	0
Pharmacist	1	1 (1)	1 (1)**[1]**
Dermatology nurse	3	0	1 (1)
Dermatology consultant	1	0	0
Allergy consultant	1	0	0
School nurse	1	0	0
Nursery manager	1	0	0
Eczema charity representative	2	0	0
**Total**	**41**	**5**	**7**

a *(round brackets denote the number who also took part in an interview), [square brackets and in bold denote number who also took part in focus group]. GPwSI *= *GP with a special interest.*

### The challenges of managing eczema

#### Lack of support and information

Parents voiced frustrations at feeling unsupported by GPs in terms of treatment, and felt their information needs were not met. The absence of specific verbal advice on how to apply emollients and TCS in terms of when, where, how often, and how much was either absent or unclear, leading to uncertainty with treatment application:

‘It was just, stick it on his legs. It really was just a kind of “here you go, this is it, and off you go”.’

(Parent [P]10)

GPs reported not providing written information to patients with eczema regularly. If they did, it was usually a generic leaflet printed from their clinical system or website. When individualised guidance was given, it tended to be in an *ad hoc* manner:

‘No, I don’t tend to give written information unless I think, “OK, this person really doesn’t know much about what they’re doing,” In which case, I might print out the patient information leaflet from Patient UK.’

(GP4)

#### Views about causes and management

Several parents held views about the causes and best method of treatment for eczema that differed from the approach expressed by healthcare professionals. Some parents believed eczema had a root cause, such as an allergy, which needed to be identified and avoided, rather than it being an inherent condition that needs to be managed with emollients and TCS. GPs recognised this issue, which they said got in the way of successfully treating and managing the condition:

‘I think they expect that, if they can find the one trigger for the eczema, they can make it all magically go away, so you have to unpick that.’

(GP14)

‘They’re going to give me the same thing they’ve been giving me for about a year, so it doesn’t really make any sense if there’s nothing to cure my daughter of the eczema.’

(P9)

#### Documenting treatment preferences

Many parents and healthcare professionals agreed that treatment tended to be on a trial-and-error basis. However, finding a treatment regime that works for the family is difficult because parents reported difficulty in remembering names of treatments tried. Also, the reasons for stopping a treatment are not routinely recorded by GPs, meaning failed treatments may be re-issued:

*‘It’s* [name of treatment] *in the notes, but what you get is, it’s unclear whether the treatments worked and they stopped using them, or it hasn’t been effective and they’ve never used it again.’*

(GP16, FGD1)

### Eczema WAP acceptability, format, and content

Box 2 gives an overview of what participants wanted from a WAP. However, differing opinions on its format and content were commonplace.

Box 2. **Overview of WAP preferences**

**Table:** 

**What should a WAP contain?** Individualised action steps for maintenance and flares When to seek medical advice Basic general information - Eczema pathogenesis - Rationale for emollients and steroids - Triggers and irritants - Recognising flares and infection Record of treatment preferences Signposting to further information **What should a WAP look like?** Ideally no more than one to two A4 pages Individualised actions on the front and general information on the back Visually appealing, with a balance of text and pictures Electronic and printed formats **WAP format** Needs to be easy to access/print/populate and, for GPs, ideally integrated into their clinical systems

*WAP* = *written action plan.*

#### Benefits of a WAP

Participants were generally positive about the role that WAPs could play in addressing barriers to eczema management. Potential benefits identified included a documented treatment plan, patients and carers empowered and confident to use treatments, an eczema information resource, and improved clinical outcomes.

#### Individualised action steps

Participants wanted a treatment plan that was individualised and specific. Also, plans should be presented in a stepped approach, that is: step 1, what to do when the condition is stable; step 2, what to do when there is a flare; and *‘how to get between the two’* steps (paediatric allergy consultant). Early interviewees who were shown the Hillingdon and Asthma UK example WAPs that take this approach thought they were a good model to follow:

‘I really liked that they had four, you know, they had sections on when the skin was kind of manageable, and what to do just to kind of maintain the skin, and then when there was actually a flare.’

(Eczema charity representative 2)

All groups wanted an additional third step indicating action to take if the condition does not respond to advised treatment:

‘There’s not a lot of support from doctors, and it’s hard to know when to take him back. I don’t want to be like a fussy parent, “oh, they’re back again with a bit of eczema”, but like I said, you know, it’s his skin.’

(P3)

Participants agreed that the WAP should be specific about what to use and when, including volume, frequency, and duration. It was also suggested that web links to videos demonstrating how to apply treatments be added.

Some also felt that providing space for a treatment log to document parents’ experiences would help either prevent re-prescribing of failed treatments, or facilitate re-ordering a successful treatment:

‘If I ring up the doctors and say “can I get another, you know, that one”, I couldn’t remember the name of it, so I was like “hang on, I’ve got to find the thing”, and have to go and rummage. So, yeah, if I had it all on there, I’d probably keep that on my fridge or something, and it’d just be all there then, in front of me.’

(P3)

‘What I like, from my perspective as a GP, is the log of previous treatments tried. It’s really common that you ask what’s been used, and it’s difficult to know because the creams look the same, they have names that are unfamiliar and, really, why would people remember unless they’ve logged it down?’

(GP17, FGD1)

Generally, this was described as a log completed by the parent, rather than the clinician.

#### General information about eczema

Participants also wanted the eczema essentials (such as generic educational information about the nature and chronicity of eczema, the rationale for emollients and TCS, with instructions about how to apply topical treatments, and reassurance and guidance on TCS use) to allay parental fears.

For TCS, healthcare professionals felt it important to explain the fingertip unit (a method of measuring TCS, where one fingertip covers an area the size of two palms):^19^

‘The fingertip full thing’s quite good, I find. That’s what I quite often say to people, “a fingertip full for this crease”, and if that could be written down, then it’s clear.’

(GP8)

Parents, notably, also wanted non-medical information on how to stop their child scratching, as this was often described as one of the most distressing behaviours related to eczema:

‘J is constantly scratching, like, even in his sleep he does it. He obviously doesn’t know he’s doing it. It would be nice if there was a way to, like, stop him doing that.’

(P3)

Another suggestion was to include a reminder about re-ordering creams, as a delay in ordering repeat prescriptions was identified as a common problem.

#### The format of the WAP

In terms of format, most parents wanted a paper copy of the WAP to display around the house. Others, *‘in an ideal world’* (P7), would appreciate an electronic copy as well, which would facilitate sharing — such as e-mailing it to school — and retention, as a single paper copy is easily damaged or lost. GPs were focused on ease of access and speed of use. The prevailing opinion was that they wanted the tool to be embedded in their clinical system and pre-populated as much as possible, for example, with patient details and treatments:

‘If it could somehow be integrated into the software of the GP consultation … we have to make some notes in the records, so, if in some way when we’re saying to a patient “put the emollient on morning and the evening and the steroid on in the middle of the day”, or whatever, that typing in types into the form that you can just print off with the one click.’

(GP9)

Once agreed and completed electronically, GPs envisaged it being saved in the medical records, and a printed copy given to the patient (and e-mailed if possible).

The draft WAP had the individualised action steps on the front page, because parents wanted something they could refer to *‘on the fridge*’ (P7), or *‘on a wall’* (P5). A traffic light colour coding system, to highlight the different steps, was also favoured: green for clear skin, amber for a flare, red for seek help. GPs cautioned that, as most surgeries do not currently have the resources to print in colour, the WAP needed to be usable in black and white as well.

### Finalising the WAP

The interviews identified a tension between having enough information to guide and support the parent, but not so much that it overwhelms or puts off the user. The first focus group felt the balance between these elements was not right in the authors’ draft WAP:

‘I’m a bit overwhelmed in terms of the amount on the page, or the amount in each box. I don’t know why, but that’s my — I can see myself handing this to someone … and then it would have to take time to explain what it all means.’

(GP16, FGD1)

The authors modified the WAP to make it less detailed and, based on the suggestion of the focus group, moved video links to demonstrations of treatment application from the back ‘information page’ to the front ‘action page’.

Subsequent participants felt that this invited users to look at them:

‘I think they look central and they look important, simply because there’s not much other information. It makes you think that these videos will have important things.’

(GP17, FGD2)

This first focus group also helped to reconcile varying views on whether the WAP should be aimed at older children (between 7–12 years old) as well as parents. The group agreed that the authors should adopt a more pictorial approach, because pictures were seen as important, not only for engaging children, but also in terms of simplifying the action steps, and overcoming language and literacy barriers:

‘I think images are sometimes more powerful … most people can, you know, this is the step and this is the arrow so you use this much, and then if it doesn’t improve, then you use — and you keep following the arrows until you get to where you want to be.’

(Pharmacist 1, FGD1).

It was not possible to include a treatment log and keep the document to two sides of A4. GPs were sceptical that parents would have the time or inclination to complete it. This view also emerged in the second focus group. However, it was agreed that, although parents may not complete it at home, it may trigger conversations about treatment acceptability during eczema consultations:

*‘Within a couple of weeks you’re saying “oh my gosh, I can’t stand my child being so greased up, I’m stopping using the hydromol, it’s a right pain”, and maybe you don’t remember to write that down. But then, almost the next time you go back to the doctors, and you’re taking this with you, and … you’re only looking at the four things* [four treatments written in the log in the WAP]*, and the doctor’s like, “well, do you want the repeat of those?” And you go “oh, hang on; we’ve stopped using that one ‘cos I hate it”.’*

(P13, FGD2)

**Figure 2.**
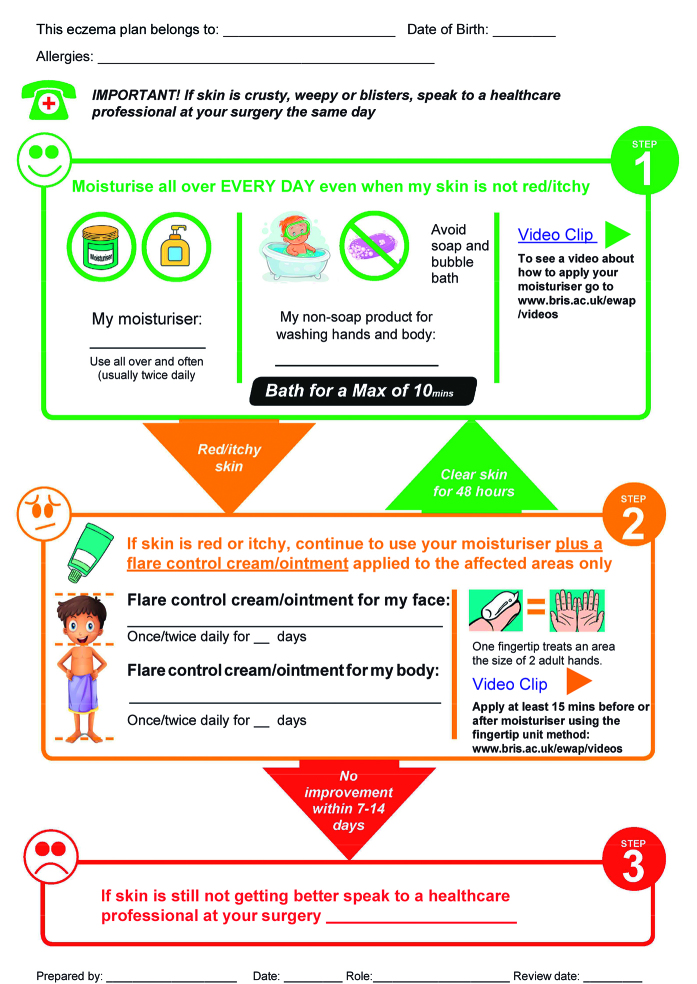
***Page 1 of the final eczema written action plan (WAP) (© University of Bristol, 2017). For further information and to download a copy of the Eczema WAP, visit http://www.bristol.ac.uk/ewap.***

Because of its potential to promote the discussion and documentation of parents’ treatment preferences, the log was included in the final version. The final WAP, excluding the treatment log, is shown in Figure 2.

## DISCUSSION

### Summary

The management of eczema in children is often impeded by parents being confused about the treatment plan. They often felt that they had received insufficient advice and information from primary care. Healthcare professionals highlighted parental belief around eczema causation leading to differing treatment expectations. All participants noted problems around the documentation of treatment preferences.

A patient-held, written self-management plan that provides clear and simple individualised treatment guidance and educational information was seen as a means of overcoming these barriers. The authors developed an eczema WAP based on the views and preferences of parents and healthcare professionals. Consistent views about format and content enabled the authors to develop draft WAPs. Focus groups helped in finalising the WAP in relation to balancing the level of detail needed for effective self-care while ensuring users are not overwhelmed with information, deciding whether the target audience should include children as well as parents, and weighing up the value of including a log of treatments.

The final, three-page eczema WAP comprises a stepped approach to treatment (maintenance, flare, seek help), eczema essentials (general information about eczema and its treatment), and a parent- completed log of previous treatments and patient preferences. It favours brevity over detail and aims to minimise completion time for healthcare professionals. Pictures are used to engage children and aid communication and understanding.

### Strengths and limitations

As far as the authors are aware, this is the first qualitative study with a range of stakeholder perspectives investigating the potential value and desired content and format of a WAP on eczema. A breadth of viewpoints, drawing on interview and focus group data, has enabled the authors to develop a user-led WAP. Having clinical and non-clinical research team members aided reflexivity during discussions throughout data analysis. The unbalanced ratio of healthcare professionals to parents in the first focus group (4:1) may have been intimidating for the lone parent and could have resulted in a biased discussion. This was countered, however, by the facilitator, who ensured all voices were heard, and the balance (4:3) in the second group was more equal. The voices in this study are predominantly those of GPs and parents, rather than those of secondary care clinicians and stakeholders, with a focus on children with eczema aged <12 years. This reflects the authors’ decision to focus on this age group/disease of mild–moderate severity, because they both represent the majority of children managed in the primary care setting. The authors sought views from the other groups, primarily to check acceptability and content of a WAP from their viewpoint, but it is possible that they failed to identify relevant opinions from these other potential users.

### Comparison with existing literature

The difficulties in treating eczema in children highlighted by this research reflect the findings of work previously undertaken with parents and carers. This includes dissatisfaction with, and confusion about, treatment plans,^8^ and the desire to find a cure rather than just maintain control.^20^ The authors also found the trial-and-error approach is hampered by parents’ struggle to recall names of treatments, and the lack of documentation about their treatment preferences in the medical notes.

National Institute for Health and Care Excellence guidance on eczema treatment highlights the importance of parental education to improve adherence and outcomes.^1^ These results support this but the findings suggest that, for any treatment plan to succeed, parental beliefs about the causes and management of eczema must be addressed, and the authors sought to achieve this by including key information about eczema in the WAP.

One of the difficulties the authors faced was how to include all of the content desired while still making it visually appealing and user friendly. A previous study looking at the readability and suitability of asthma WAPs exposed some of the common pitfalls: too many check boxes for medications and doses, lack of visual cues, such as boxes and arrows, practitioner-centred wording, and lack of white space.^21^ Some of these issues featured in an earlier draft of the authors’ WAP, but the readability and usability were evaluated by participants at each stage of its development, improving the final product.

A qualitative systematic review looking at the design and use of WAPs in asthma and eczema depicted a preference for pictures for universal communication.^22^ A randomised trial also found a pictorial WAP was preferred by healthcare professionals, compared with a standard WAP, and provided clearer communication for self-management and treatment.^23^ These findings also support the value of including pictures for engaging children and parents in managing eczema.

### Implications for research and practice

The authors have developed a WAP for children with eczema, using input from a wide range of parents, clinicians, and stakeholders. The draft WAP has been improved by a professional designer without changing the core aspects identified by the study participants. Further research is needed to trial the WAP in practice, to establish whether it can empower parents, children, and healthcare professionals to improve the management and treatment of childhood eczema in primary care.
